# A Tanzanian Boy with Molecularly Confirmed X-Linked Adrenoleukodystrophy

**DOI:** 10.1155/2019/6148425

**Published:** 2019-12-31

**Authors:** M. C. J. Dekker, A. M. Sadiq, R. Mc Larty, R. M. Mbwasi, M. A. A. P. Willemsen, H. R. Waterham, B. C. Hamel

**Affiliations:** ^1^Department of Paediatrics and Child Health, Kilimanjaro Christian Medical Centre, P.O. Box 3010, Moshi, Tanzania; ^2^Department of Radiology, Kilimanjaro Christian Medical Centre, P.O. Box 3010, Moshi, Tanzania; ^3^Department of Pediatrics, Radboud University Medical Center, Geert Grooteplein Zuid 10, 6525 GA Nijmegen, Netherlands; ^4^Laboratory Genetic Metabolic Diseases, Amsterdam UMC—Location AMC, University of Amsterdam, Amsterdam, Netherlands; ^5^Department of Human Genetics, Radboud University Medical Center, Geert Grooteplein Zuid 10, 6525 GA Nijmegen, Netherlands

## Abstract

Adrenoleukodystrophy (ALD) is an X-linked peroxisomal disorder with classical features, which can be also recognised in a low resource setting. It had been described in various populations across the globe, but very few cases have been reported from Africa. In a boy with features of a progressive central nervous system condition and adrenal failure, *ABCD1* gene screening was performed based on a clinical history and basic radiological features which were compatible with ALD. A common *ABCD1* mutation was identified in this patient, which is the first report of genetically confirmed ALD in Sub-Saharan Africa. ALD is likely under recognised in those areas where there is no neurologist. This genetic confirmation widens geographical distribution of *ABCD1*-associated disease, and illustrates recognisability of this disorder, even when encountered in a low-resource environment.

## 1. Introduction

X-linked adrenoleukodystrophy (ALD, OMIM #300100) is the most common peroxisomal disorder in the world [1, 2], and the most frequent genetic disorder affecting myelin present in the central and peripheral nervous system. The main biochemical abnormality is the accumulation of very long-chain fatty acids (VLCFA) in tissues and body fluids subsequent to their defective catabolism in the peroxisomes. ALD results from a deficiency in *ABCD1*, ATP-binding cassette sub-family D, member 1, also called ALDP (adrenoleukodystrophy protein), which is encoded by the *ABCD1* gene on the X-chromosome [3]. A predictable genotype-phenotype relation is lacking. There is great diversity of mutations and degrees of biochemical abnormalities versus clinical expression. Furthermore, disease penetrance is not 100%: 5–10% of mutation carriers have other presentations or are asymptomatic [4].

The principal characteristic of the disease is the concurrent combination of a neurological and an endocrine disorder, the latter being primary adrenocortical insufficiency, and occasional testicular failure. There are two main clinical presentations, the so-called “childhood cerebral form” with (sub) acute onset, and rapidly fatal cerebral involvement in young boys (35% [4]), and the other form generally affecting young men with slowly progressive myelopathy and peripheral neuropathy (adrenomyeloneuropathy (AMN)) (40–45% [4]). Adrenal insufficiency is another clinical phenotype and can stand alone or occurs before, after or at the same time as neurological disease (10% [4]). Its occurrence does not predict the severity of the neurological disorder. Conversely, ALD is a differential diagnostic consideration in any male patient with primary adrenal insufficiency. A relatively large proportion of female *ABCD1* mutation carriers develops a slowly progressive myelopathy too [5]. Bone marrow transplantation is indicated in young children with ALD who show early evidence of cerebral involvement, but in settings where this is unavailable such as most of the Sub-Saharan Africa (SSA), the cerebral form is rapidly lethal.

Mutations in *ABCD1* have been described in ALD patients from populations around the world [1], but reports from Africa are limited to a few North African countries. In the Republic of South Africa ALD does occur [6–8] although genetic confirmation is not available in scientific literature.

## 2. Case Report

**Figure 1:**
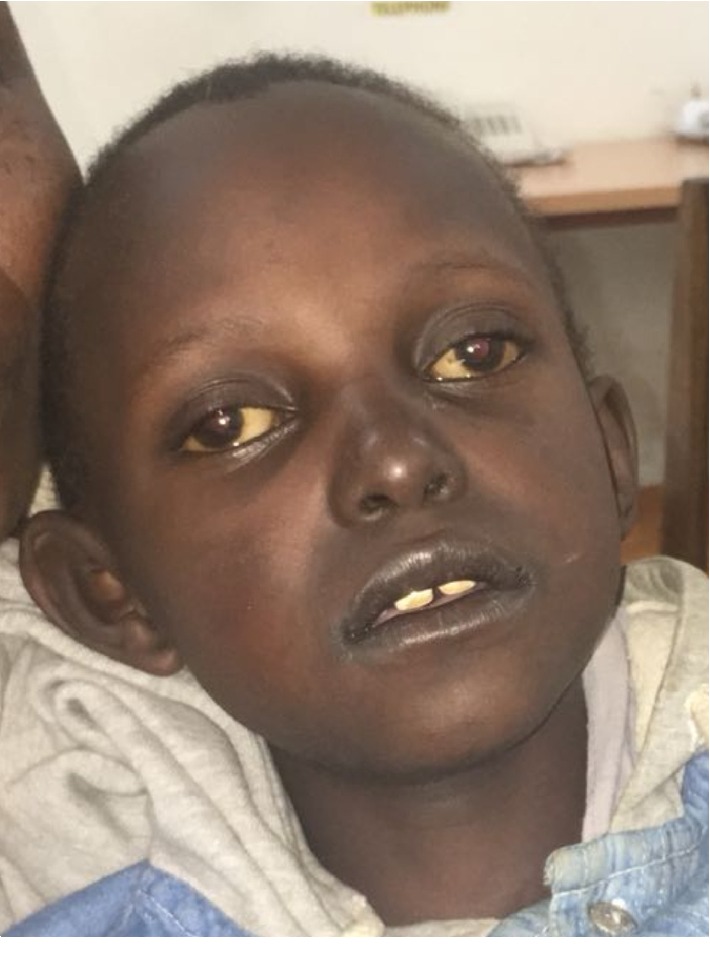
Slate-grey appearance of face.

**Figure 2:**
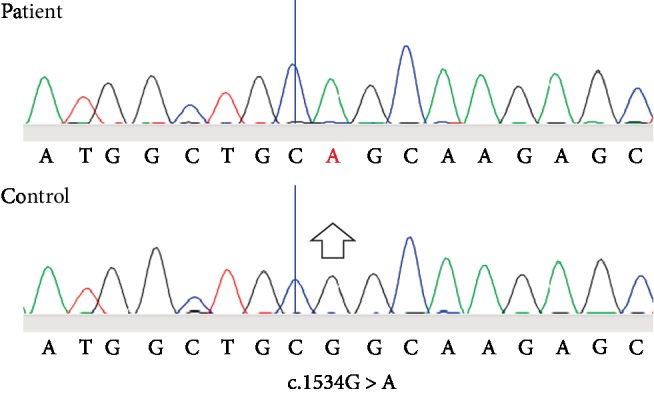
Electropherogram.

We present a 9-year-old male, single child from nonconsanguineous parents from Northern Tanzania. The father was not living with the mother, but was reportedly well. The mother's gait was normal but further neurological examination has not been performed due to loss to follow-up. There was no previous medical history. The child had normal milestones and had been performing well until recently, but has always been shorter than his age mates. He reported to our clinic at the Kilimanjaro Christian Medical Centre, a tertiary referral hospital in Moshi, Northern Tanzania with a 3-4 month history of progressive confusion and difficulty walking. There were episodes of aggression and hyperactivity which fluctuated over days and were worse at night. He had been asked to leave the school. In the same period he developed difficulty walking and had become incontinent for urine and faeces. There was no acute onset, nor was there a febrile illness preceding the complaint. Upon physical examination, we saw a proportionally small child, height 110 centimetres (below third percentile, [9]) and weight 24 kilograms (fifth percentile [9]). His nutritional condition was moderate. General physical examination, and notably the skin, was normal and there were no dysmorphic features. Head circumference was normal. Upon neurological screening, the boy appeared to be only partially aware of his environment, talking to no one in particular and acting defensively and aggressively at times. Cranial nerve exam was unremarkable. Fundoscopy of the optic nerve and retina was normal. Visual acuity testing was complicated by a short attention span. He could, however discern and localise bright and moving objects. Motor exam revealed moderate spasticity with exaggerated reflexes for as far as the child permitted examination, plantars were upgoing bilaterally and he was visibly incontinent. Proximal leg musculature was grade 5-/5, muscle bulk was slightly decreased in keeping with a short stature and moderate nutritional condition. He could walk with assistance with a broad based, high stepping gait. In the course of months upon follow-up, he developed fatigue, generalised lassitude, and a slate-grey discolouration most prominently around the nose and mouth, tongue, hand palms, and around skin scars (see Figure 1). He was always feeling cold and was too tired to eat. Laboratory investigations were normal except a hyponatremia of 116 mmol/l and hyperkalaemia which was initially treated with hypertonic saline and a salt-enriched diet, but did not improve much. HIV test was negative and chest X-ray normal. Lumbar puncture showed a normal number of cells, protein, and glucose levels. Immune electrophoresis is not available in our centre and was not affordable through a commercial laboratory. Computed Tomography (CT, Supplementary Material [Supplementary-material supplementary-material-1]) and Magnetic Resonance Imaging (MRI, Supplementary Material [Supplementary-material supplementary-material-1]) of the brain showed a striking posteriorly distributed hypodense white matter, with volume loss and enhancement with contrast on MRI. The characteristic radiological pattern with anterior sparing of extensive white matter abnormalities in combination with neurodegeneration and adrenocortical failure suggested ALD. The working diagnosis was a rapidly progressive multifocal central nervous system disorder, with in second instance an endocrine complication, query hypothyroidism or adrenocortical insufficiency. Because of the short history, initially a low- grade encephalopathy or encephalitis was suspected, with HIV as a main infection to be ruled out. Alternatively, an inflammatory demyelinating encephalopathy was queried. Acute Demyelinating Encephalomyelitis (ADEM) or auto-immune encephalitis was amongst the possibilities, for which a lumbar puncture was performed. Less likely in this SSA setting but not impossibly would this be the onset of a metabolic or neurodegenerative disorder. Adrenocortical failure was subsequently suspected based on clinical grounds, and oral hydrocortisone supplementation therapy was started. Very long chain fatty acid level, cortisol, and ACTH testing was not available in Northern Tanzania. Within days, his general condition markedly improved, but not his neurological performance. Later on, for local unavailability of oral hydrocortisone therapy, the patient had to be switched to prednisolone. This allowed for some improvement albeit not as much as before. Written informed consent for genetic testing of the *ABCD1* gene was obtained from the mother for genetic research and photographs, in Swahili and English. Venous blood was sampled from the patient, and was sent to the Laboratory Genetic Metabolic Diseases, Amsterdam UMC, Amsterdam, The Netherlands. Sanger sequencing was performed of all coding exons plus flanking intron sequences of the *ABCD1* gene. DNA sequence analysis of the *ABCD1* gene (NM_000033.3) revealed the hemizygous variant c.1534G>A (p.(Gly512Ser)) which is a recurrent, pathogenicity class 5 variant (see electropherogram in Figure 2). No additional pathogenic mutations were identified in the *ABCD1* gene; this confirms the diagnosis of ALD. The mother and grandmother of the patient were counseled about the findings but since, has not yet been available for further testing. The patient unfortunately passed away a year after the diagnosis.

## 3. Discussion

As far as our literature search allows us to conclude, this is the first genetically confirmed ALD patient in SSA. The commonly occurring c.1534G>A (p.(Gly512Ser)) mutation has been described elsewhere, and adds to data on its panethnic occurrence. There is a void on the map between Northern Africa and the Republic of South Africa which is likely explained by the lack of recognition and testing resources for ALD, rather than by the absence of the condition in these regions. It nevertheless confirms its presence in SSA, adding to the very few reports from the African continent.

There are reports on ALD or its clinical suspicion from South Africa, but cases published do not mention genetic confirmation [6–8]. The c.1534G>A (p.(Gly512Ser)) mutation causes a complete loss of function with no detection of ADLP in patient cells [10]. This specific mutation has been described before in many regions of the world especially Brazil, China, Japan, and various European countries, accounting for 32 patients in the ALD Mutation Database [11]. This dedicated database contains 22 references for this specific mutation, but no reports on African patients with this mutation are available. Four references concerned unpublished material from molecular diagnostics laboratories and newborn screening programme databases. One of the four addressees responded that no Africans with the specific mutation were in the database, but two Somalian patients were diagnosed with other *ABCD1* mutations; two colleagues responded that ethnicity had not been registered due to the genetically diverse local population; finally one molecular diagnostics laboratory is yet to respond to our query.

There are ALD patients from elsewhere in Africa, some genetically confirmed with other *ABCD1* mutations, see Table 1 for an overview. Most of those patients originate from Northern Africa [12–19]. The African origin of the patient in the United States is not known in detail [20]. Some of the ALD patients from a South African referral hospital, which included patients of mixed and African ancestry, have been genetically confirmed but details were not available (personal communication, M. Hendricks/J. Wilmshurst).

This patient presented with a rapidly progressive neurodegenerative disorder in combination with adrenal failure, in a setting without genetic or neurometabolic diagnostic facilities in a radius which measures several countries across the African continent- let alone that this specific family would have the financial resources for such testing. Imaging was performed, however, which already on a nonenhanced CT of the brain showed a very characteristic white matter hypodensity pattern. It illustrates the fact that in a setting generally devoid of higher-level testing facilities, the diagnosis can be prompted by these characteristic clinical features. It thus stresses the importance of training in potentially treatable paediatric neurological conditions. Tragically, however, such treatments are not yet available in most of SSA.

## Figures and Tables

**Table 1:** Reports on ALD from the African continent.

Country of origin	Number of patients	Clinical phenotype	Genetic confirmation	*ABCD1* mutation	Reference
Tunisia	1	ALD	Yes	c.947A>C; p.(Gln316Pro)	Kallabi et al. (2016)
Tunisia	1	ALD	Yes	c.284C>A; p.(Ala95Asp)	Kallabi et al. (2013)
Tunisia	1	ALD	Yes	c.1780+2T>G	Kallabi et al. (2015)
Morocco	8	Not elaborated	Not known	No data	Benjelloun et al. (2017a)
Morocco	9	Not elaborated	Not known	No data	Benjelloun et al. (2017b)
Morocco	1	ALD	Yes	c.1677C>G; p.(Tyr559^∗^)	Karkar et al. (2015)
Israel of Jewish Moroccan descent	3	ALD	Yes	c.1072T>C; p.(Leu229Pro)	Neumann et al. (2001)
United States of African-American descent	1			c.1489-2A>G (g.153005544)	Chen et al. (2018)
United States of Somalian descent	2	ALD	Yes	Details not known, different from mutation reported in this study	Personal communication, K. Wiens
South Africa	Various, including of mixed and African descent	ALD	Yes	Details not available	Van Eyssen et al. (2017), personal communication, Wilmshurst J., Hendricks M.
South Africa	1 (ethnicity not described)	AMN	Unknown, no response to queries		Terre'Blanche et al. (2011)
South Africa	1 (ethnicity not described)	Addison's disease mimic	Unknown, no response to queries		Soule (1999)
France of North African descent	1	Adult-onset AMN	Not possible at moment of publication		Turpin (1985)

ALD = adrenoleucodystrophy; AMN = adrenomyeloneuropathy. c.1677C > G; p.(Tyr559^∗^) is a nonsense mutation resulting into a stop codon affecting the ALDP (adrenoleucodystrophy protein).

## Supplementary Materials

Supplementary 1 Figure S1: CT images of patient. S1A to S1E: Noncontrast axial images. S1F: noncontrast coronal image. S1G to S1H: noncontrast sagittal images. Noncontrast images demonstrate symmetrical confluent hypodensity in the parietal periventricular white matter, splenium of the corpus callosum, internal capsule, and the thalamus. No mass effect on the lateral ventricles. There is presence of mild cerebral atrophy with prominence of sulci, dilatation of ventricles and cisternal spaces. Features are consistent with X-linked adrenoleukodystrophy.

Supplementary 2 Figure S2: MRI of patient. S2A-S2B: Flair axial images. S2C-S2E: T2 weighted images. S2F-S2I: T1 contrast fat sat axial images. S2J-S2K: T1 contrast fat sat sagittal images. S2L-S2M: Diffusion Weighted Images (DWI). S2N-S2O: Apparent Diffusion Coefficient (ADC maps). MR sequences show bilateral symmetrical periventricular parietal white matter lesion which is hyperintense of Flair and T2WI and hypointense on T1 C+ fat sat images with peripheral intermediate zone enhancement. The lesion extends to the splenium of the corpus callosum, internal capsule, white matter tracks in the thalamus and inferiorly to the midbrain and pons. On DWI and ADC sequences, the intermediate zone of active demyelination shows restricted diffusion. Findings are consistent with X-linked adrenoleukodystrophy.
