# Supplementary material for: A Tanzanian Boy with Molecularly Confirmed X-Linked Adrenoleukodystrophy
Source: Case Rep Genet. 2019 Dec 31;2019:6148425. doi: 10.1155/2019/6148425 (PMC7011349; doi:10.1155/2019/6148425)
Supplement: Supplementary 1 — Figure S1: CT images of patient. S1A to S1E: Noncontrast axial images. S1F: noncontrast coronal image. S1G to S1H: noncontrast sagittal images. Noncontrast images demonstrate symmetrical confluent hypodensity in the parietal periventricular white matter, splenium of the corpus callosum, internal capsule, and the thalamus. No mass effect on the lateral ventricles. There is presence of mild cerebral atrophy with prominence of sulci, dilatation of ventricles and cisternal spaces. Features are consistent with X-linked adrenoleukodystrophy. [file 6148425.f1.docx]

**Legend Supplementary Material**

**Figure S1**: CT images of patient. S1A to S1E: Non-contrast axial images. S1F: Non-contrast coronal image. S1G to S1H: Non-contrast sagittal images. Non-contrast images demonstrate symmetrical confluent hypodensity in the parietal periventricular white matter, splenium of the corpus callosum, internal capsule and the thalamus. No mass effect on the lateral ventricles. There is presence of mild cerebral atrophy with prominence of sulci, dilatation of ventricles and cisternal spaces. Features are consistent with X-linked adrenoleukodystrophy.


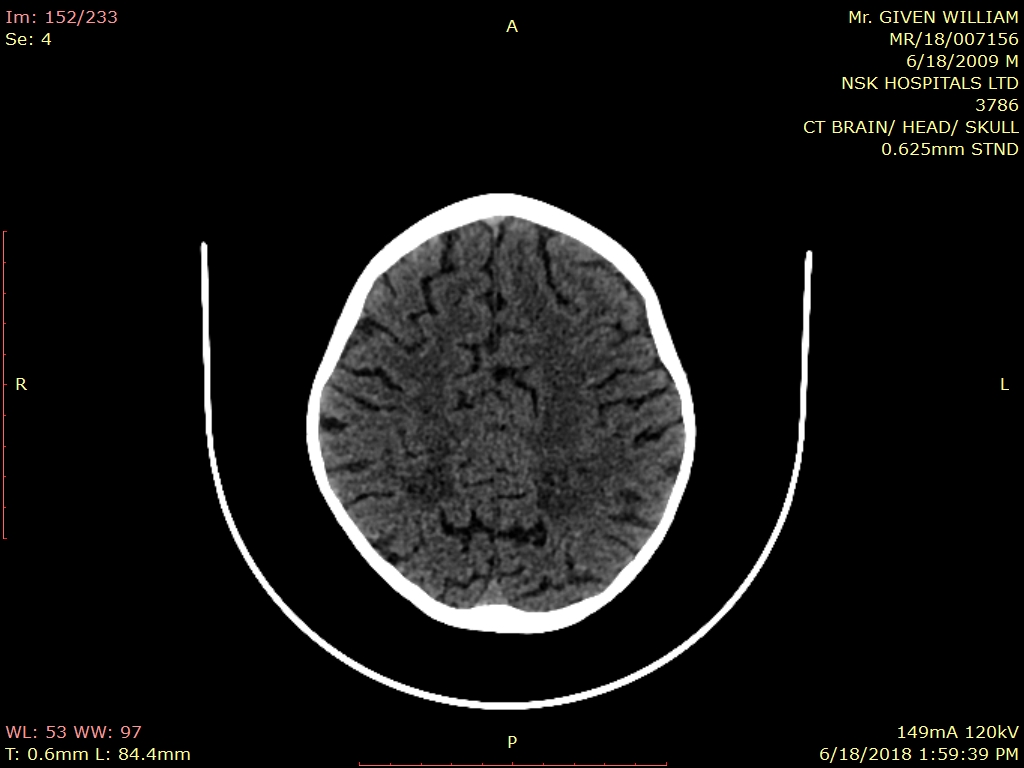

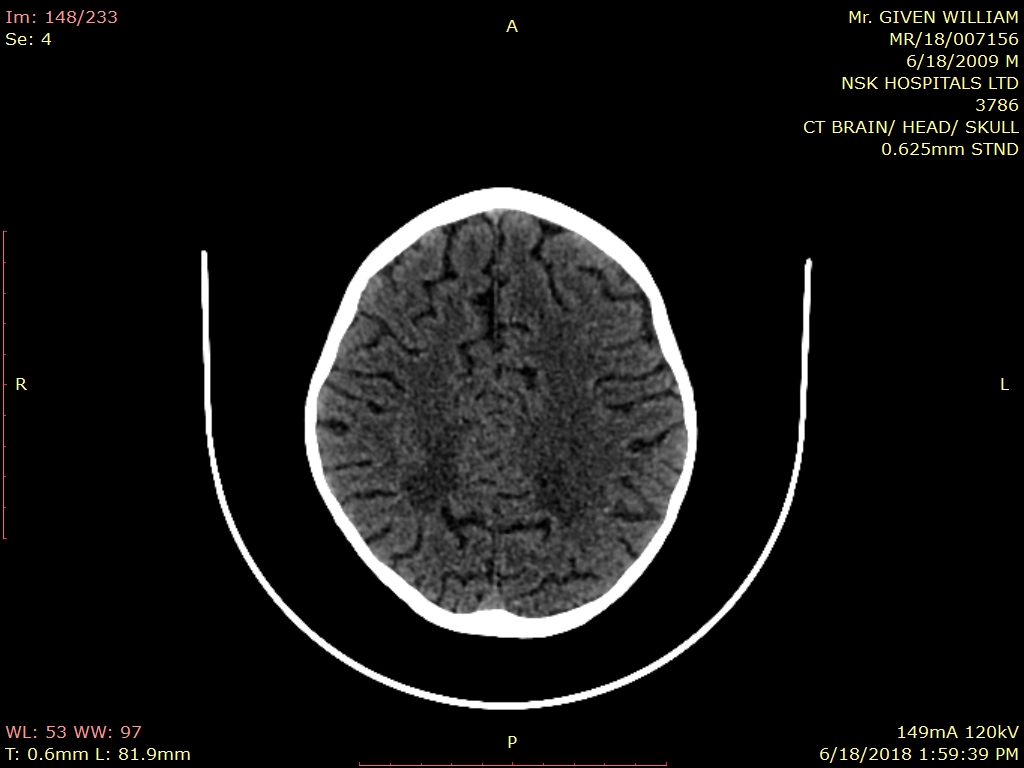

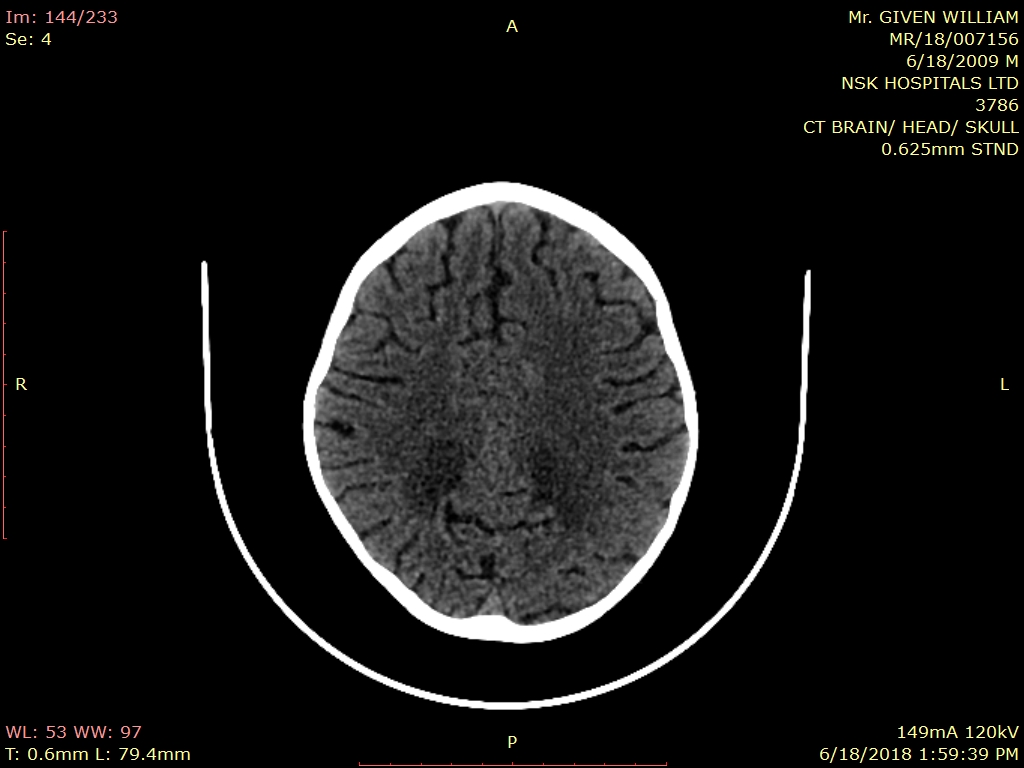

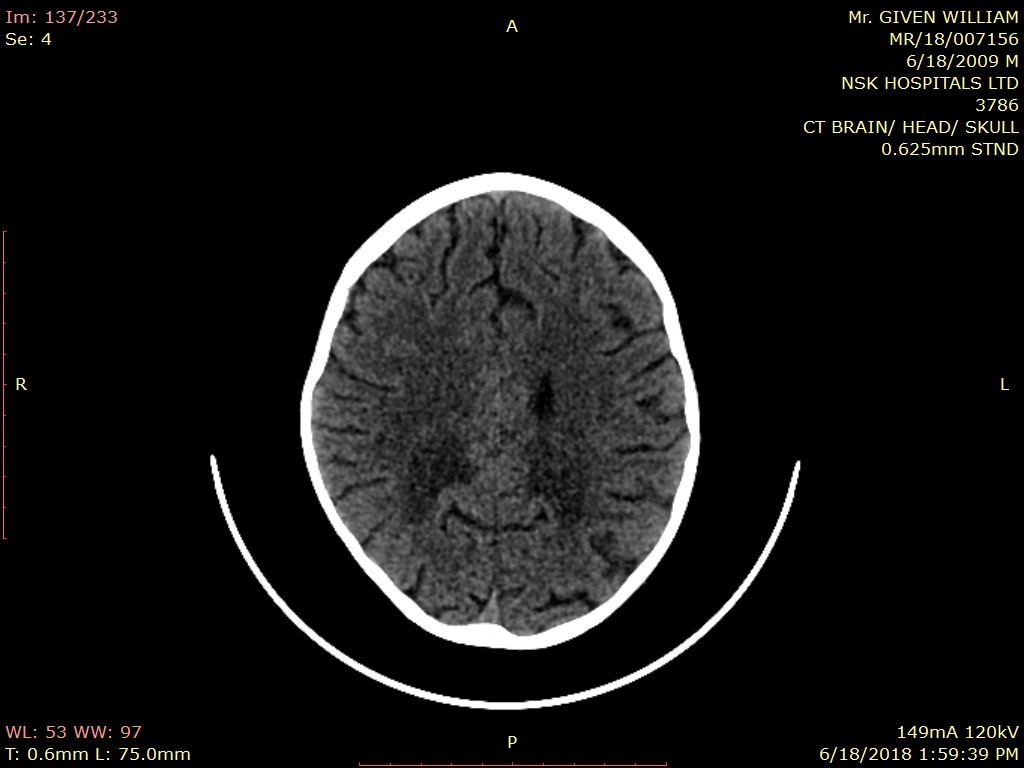


A B C D


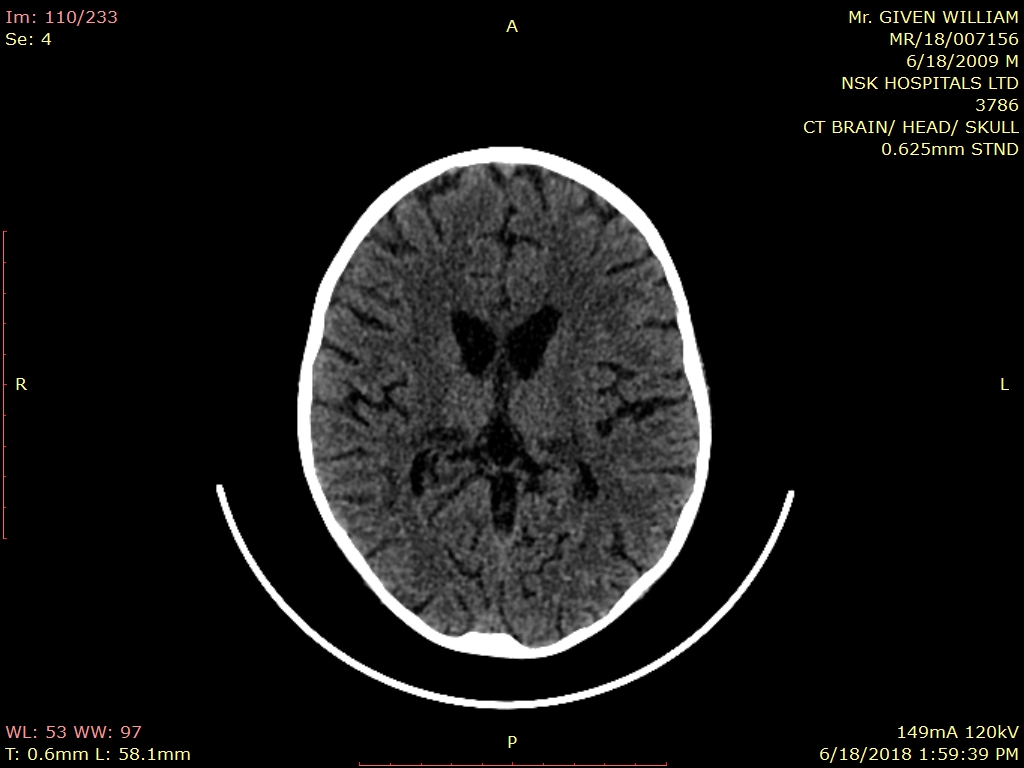

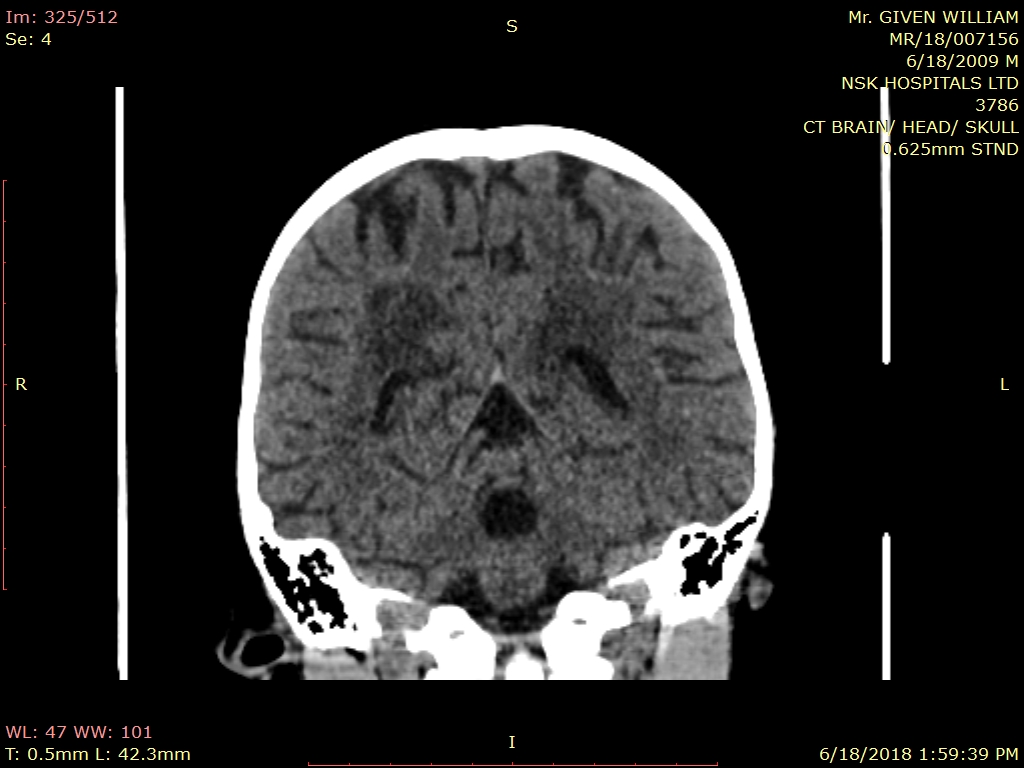

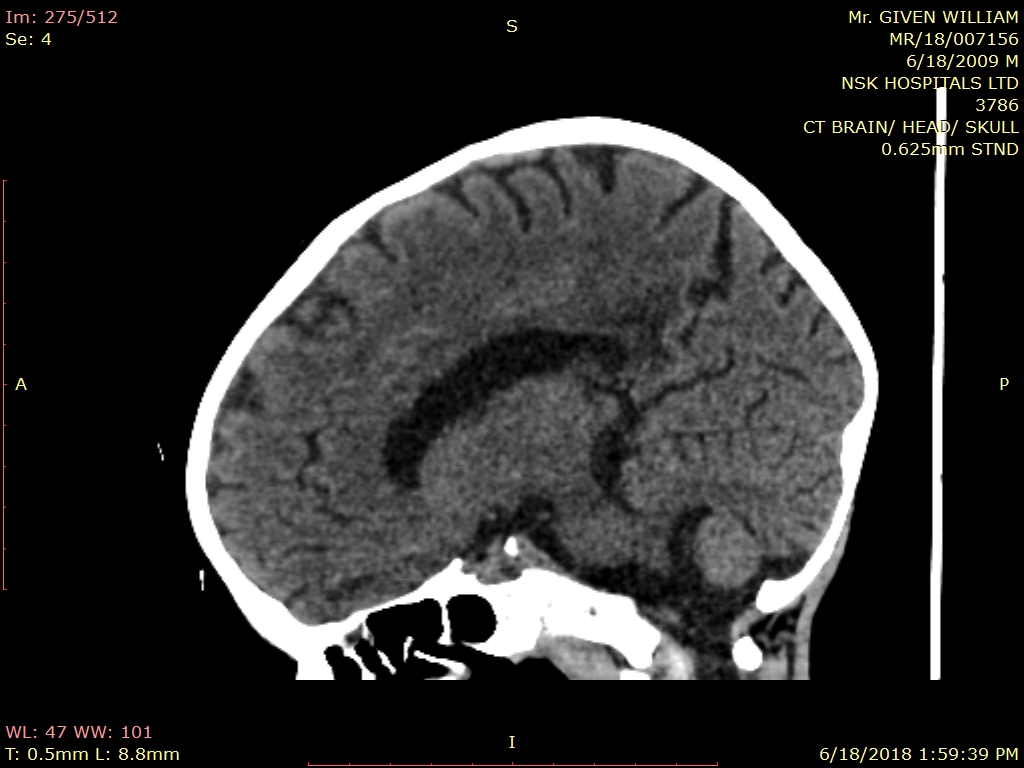

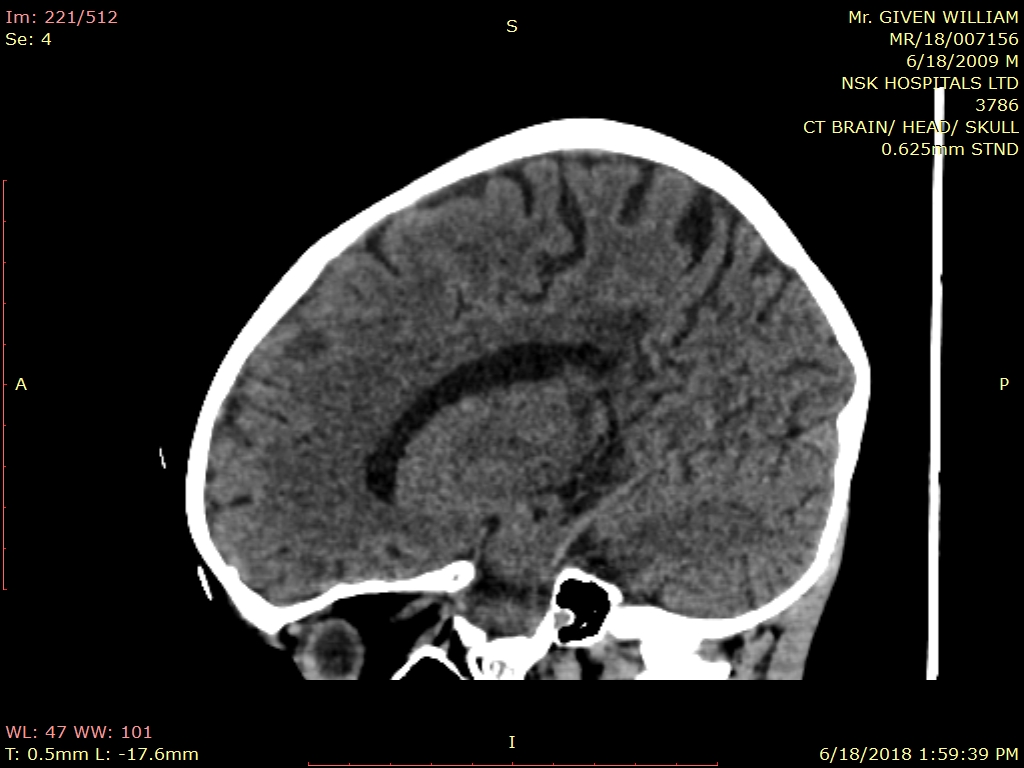


E F G H
