# Supplementary material for: A Tanzanian Boy with Molecularly Confirmed X-Linked Adrenoleukodystrophy
Source: Case Rep Genet. 2019 Dec 31;2019:6148425. doi: 10.1155/2019/6148425 (PMC7011349; doi:10.1155/2019/6148425)

**Legend Supplementary Material**

**Figure S2**: MRI of patient. S2A-S2B: Flair axial images. S2C-S2E: T2 weighted images. S2F-S2I: T1 contrast fat sat axial images. S2J-S2K: T1 contrast fat sat sagittal images. S2L-S2M:

Diffusion Weighted Images (DWI). S2N-S2O: Apparent Diffusion Coefficient (ADC maps). MR sequences show bilateral symmetrical periventricular parietal white matter lesion which is hyperintense of Flair and T2WI and hypointense on T1 C+ fat sat images with peripheral intermediate zone enhancement. The lesion extends to the splenium of the corpus callosum, internal capsule, white matter tracks in the thalamus and inferiorly to the midbrain and pons. On DWI and ADC sequences, the intermediate zone of active demyelination shows restricted diffusion. Findings are consistent with X-linked adrenoleukodystrophy.


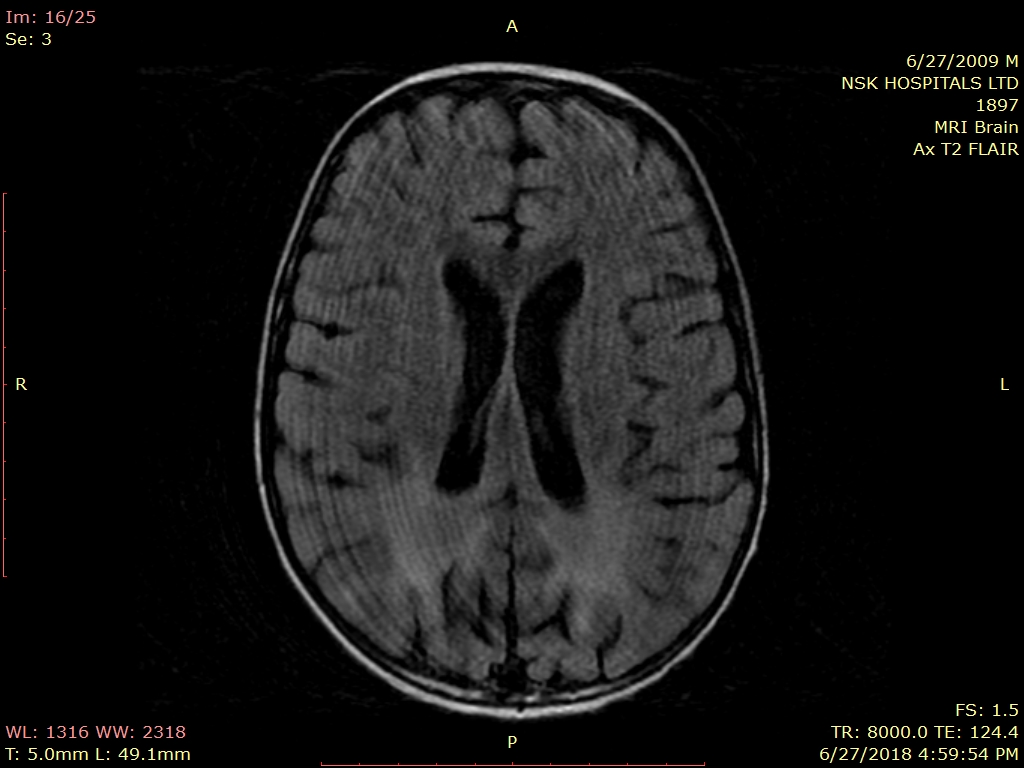

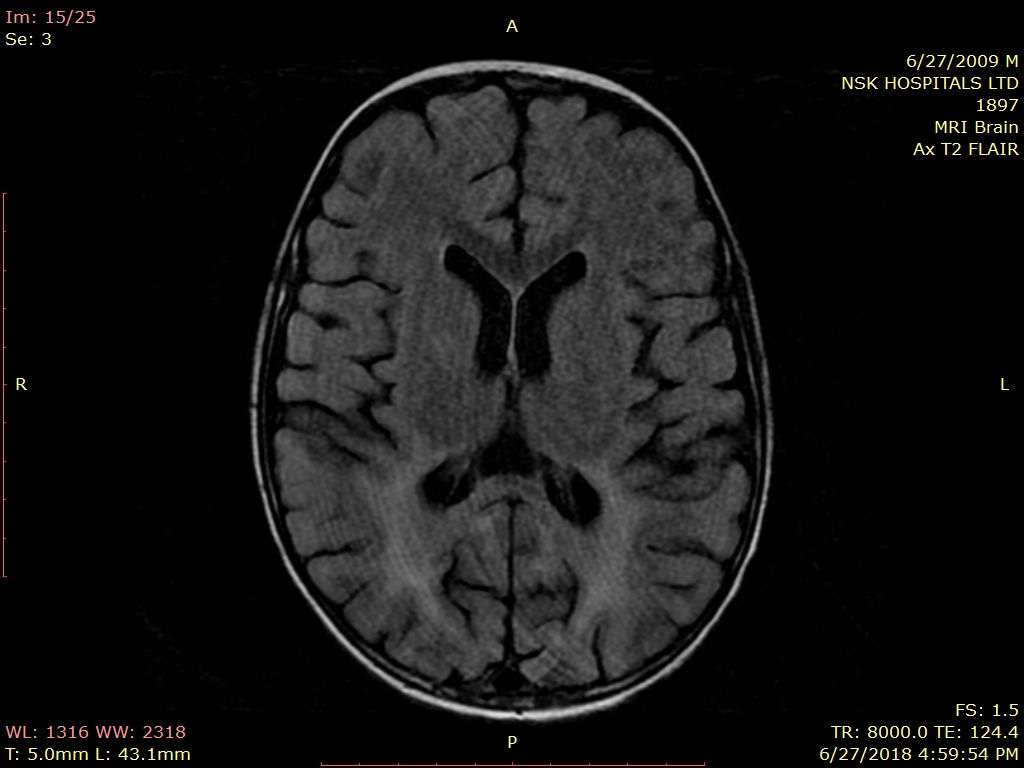


A B


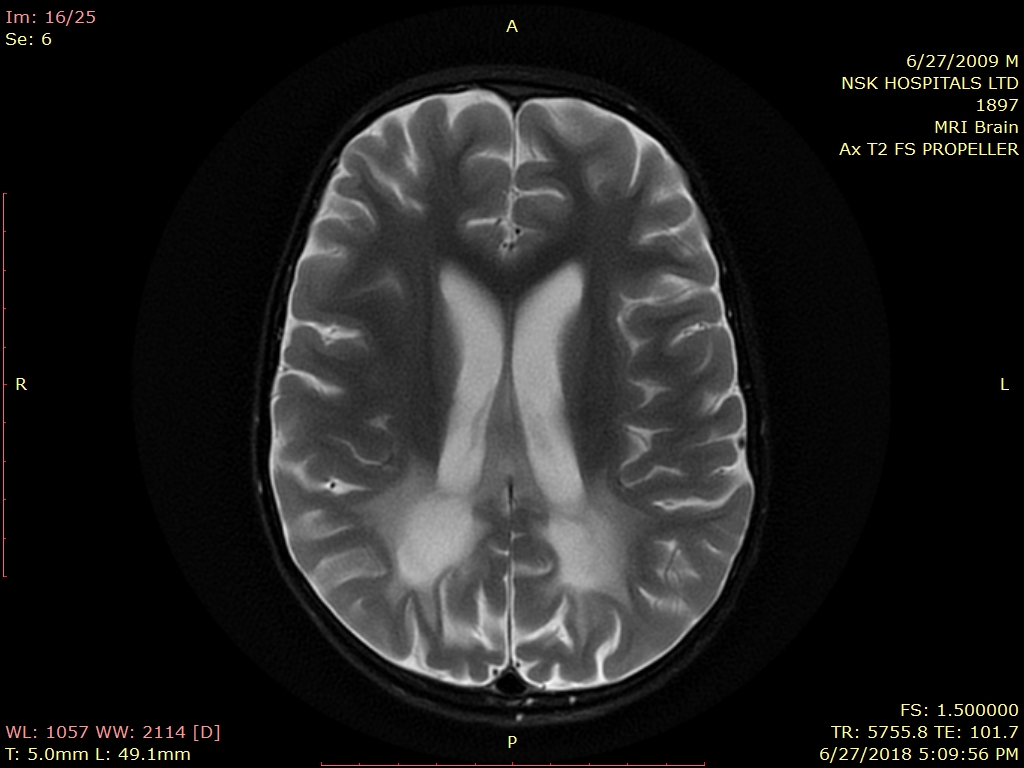
\
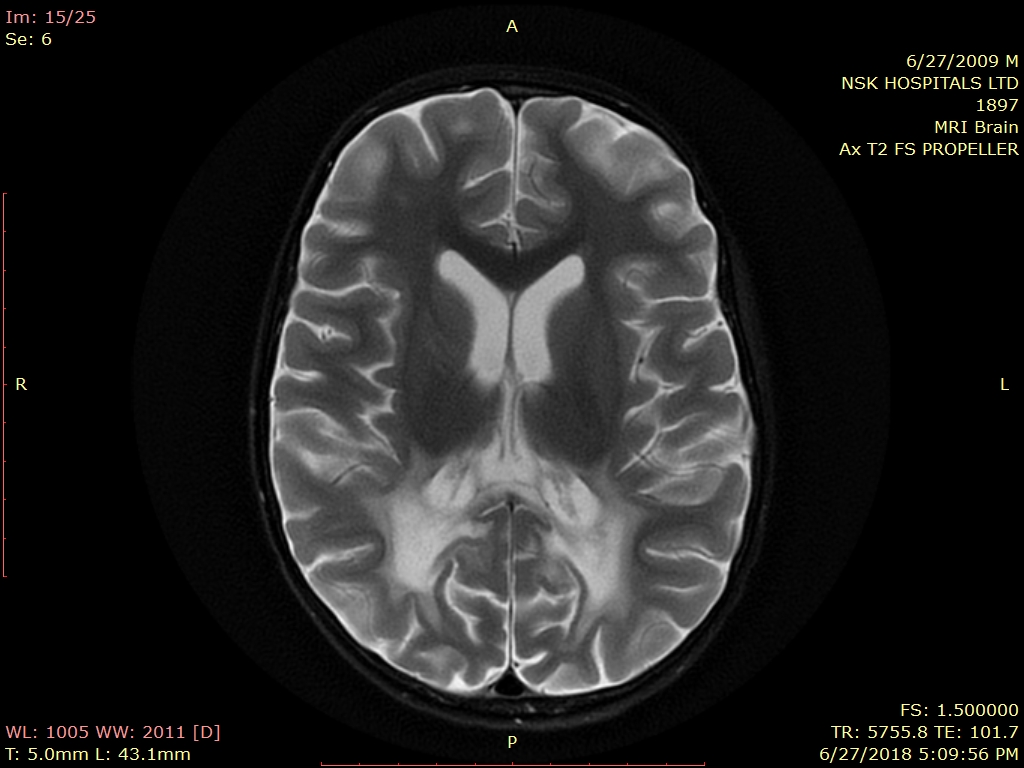

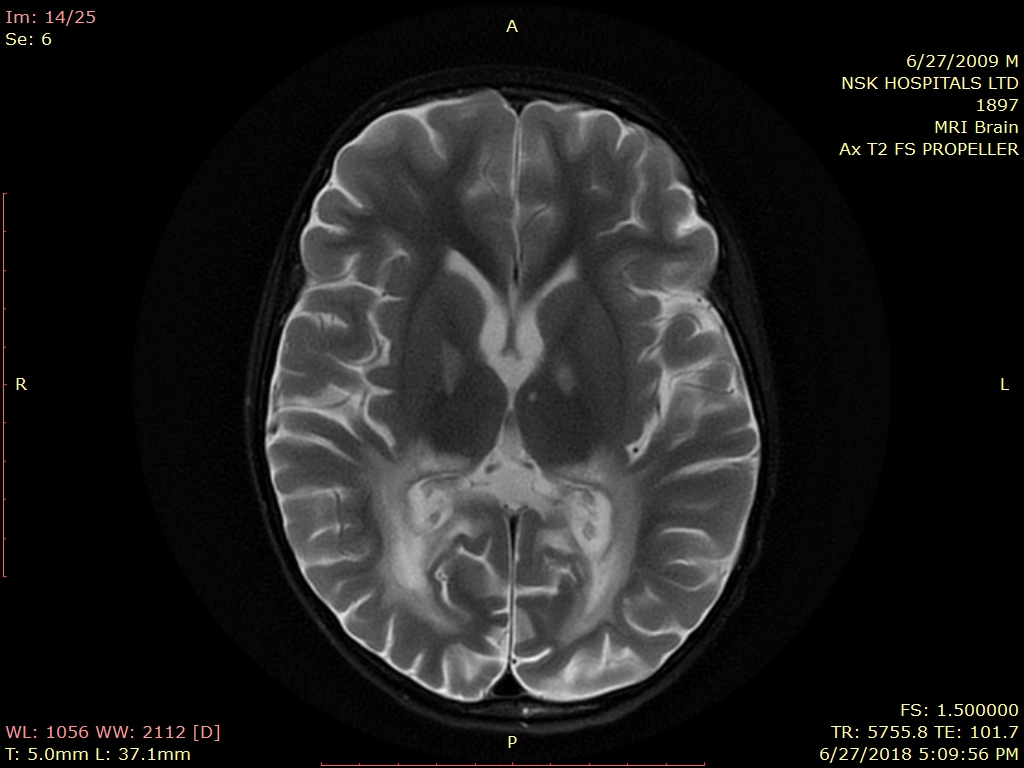


C D E


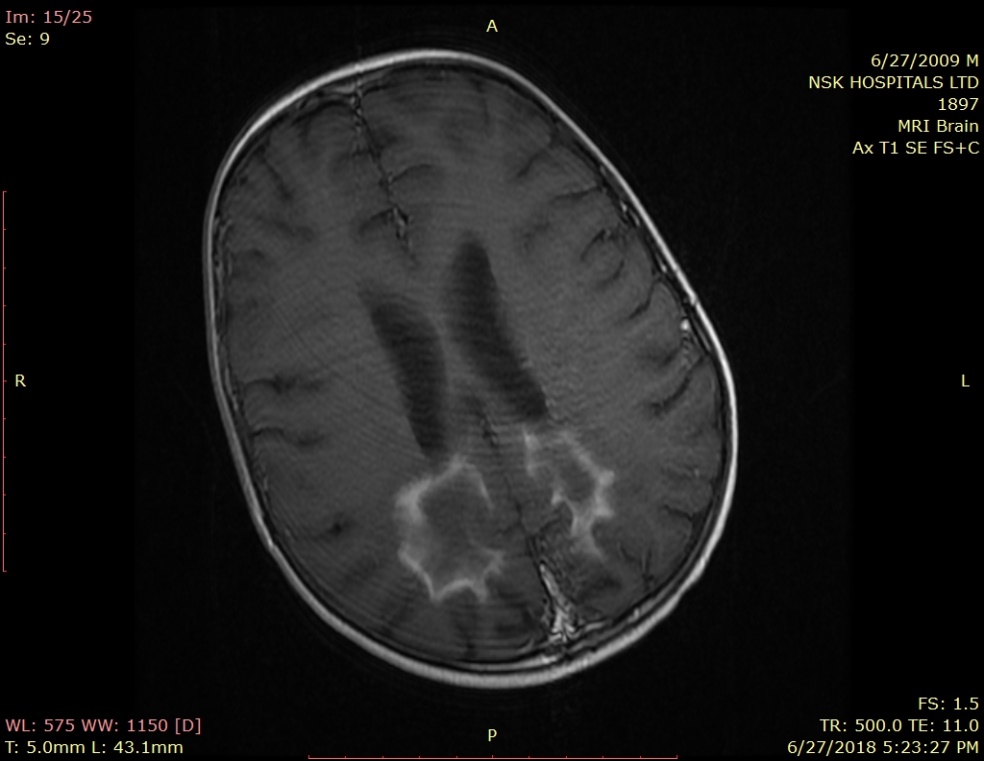

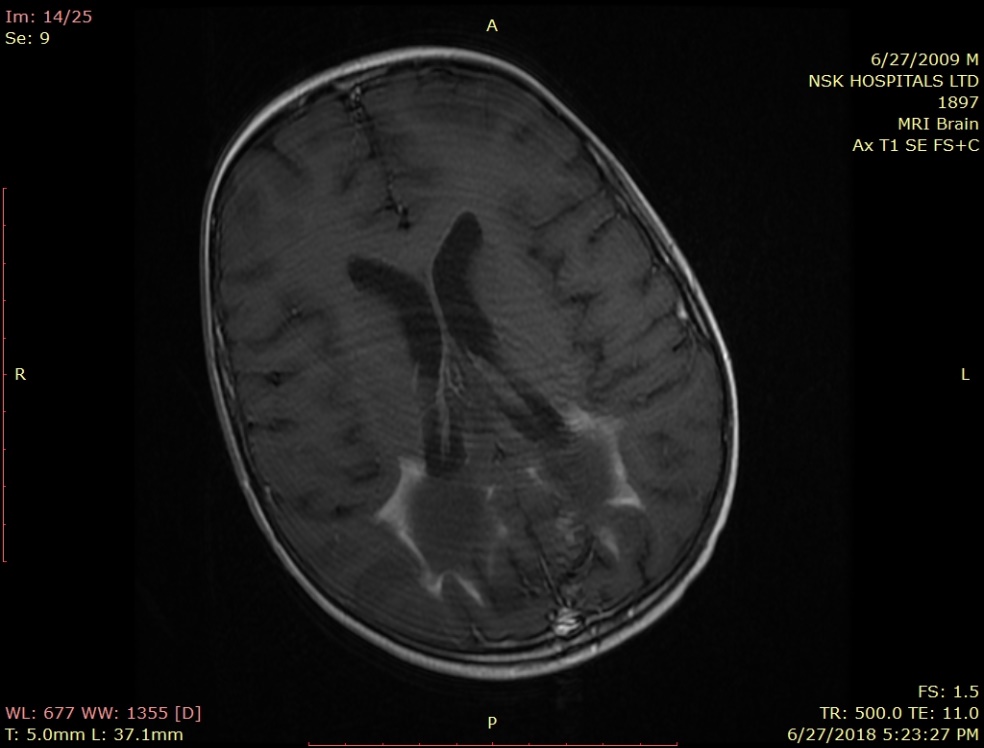

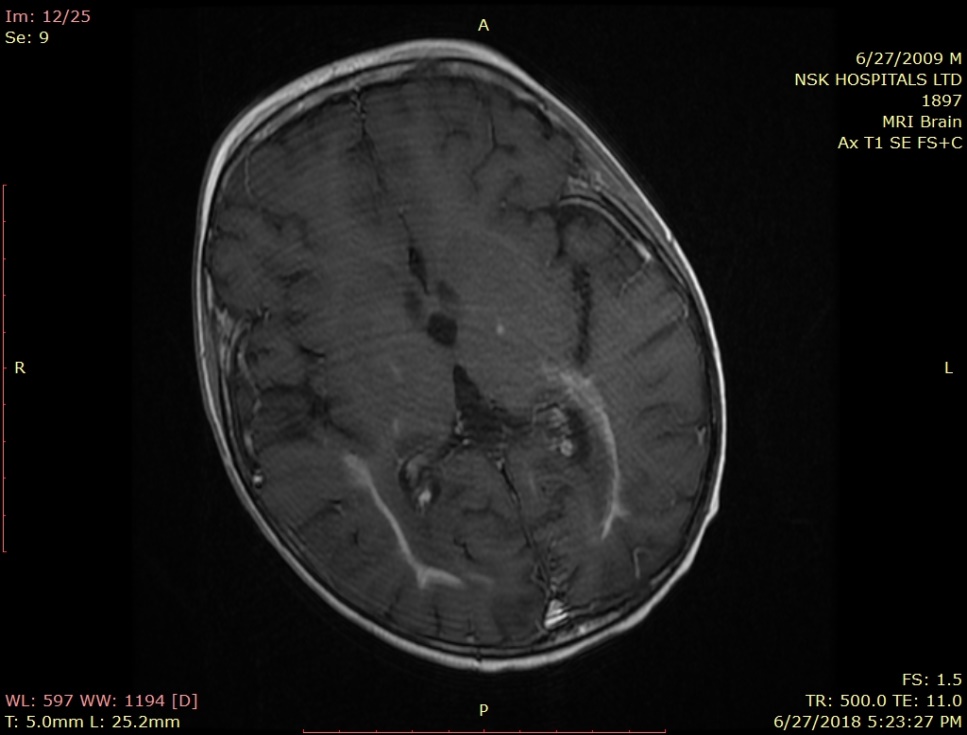

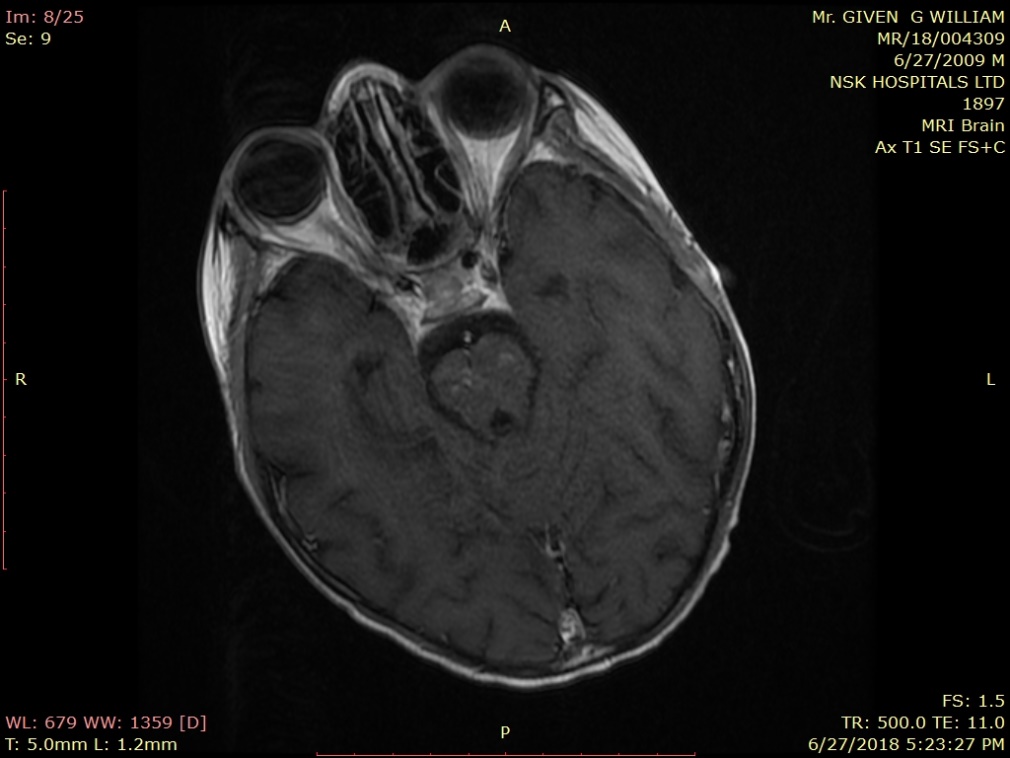


F G H I


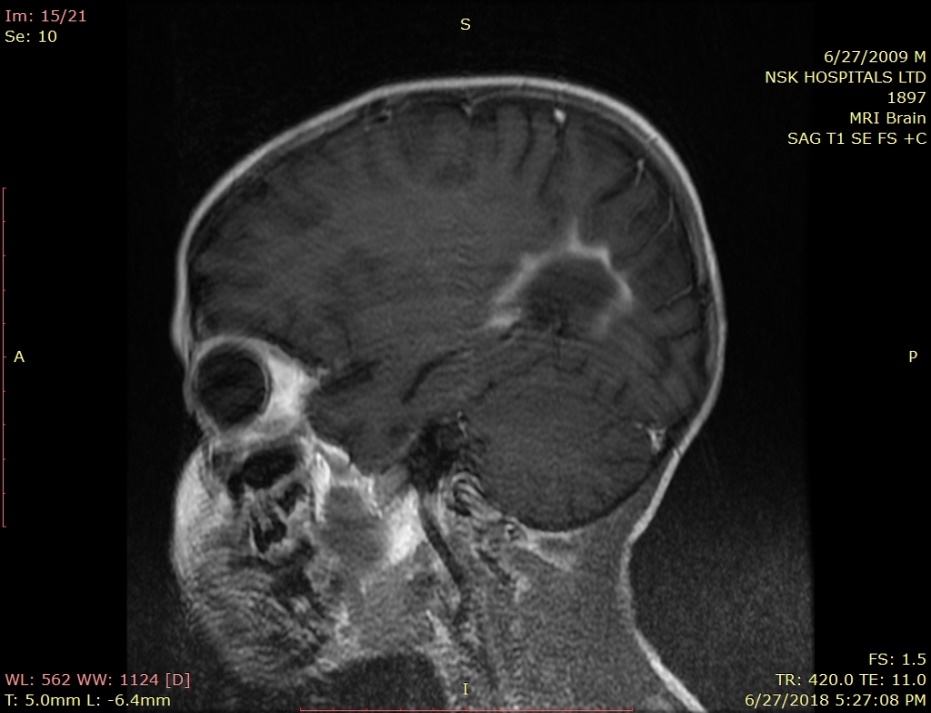

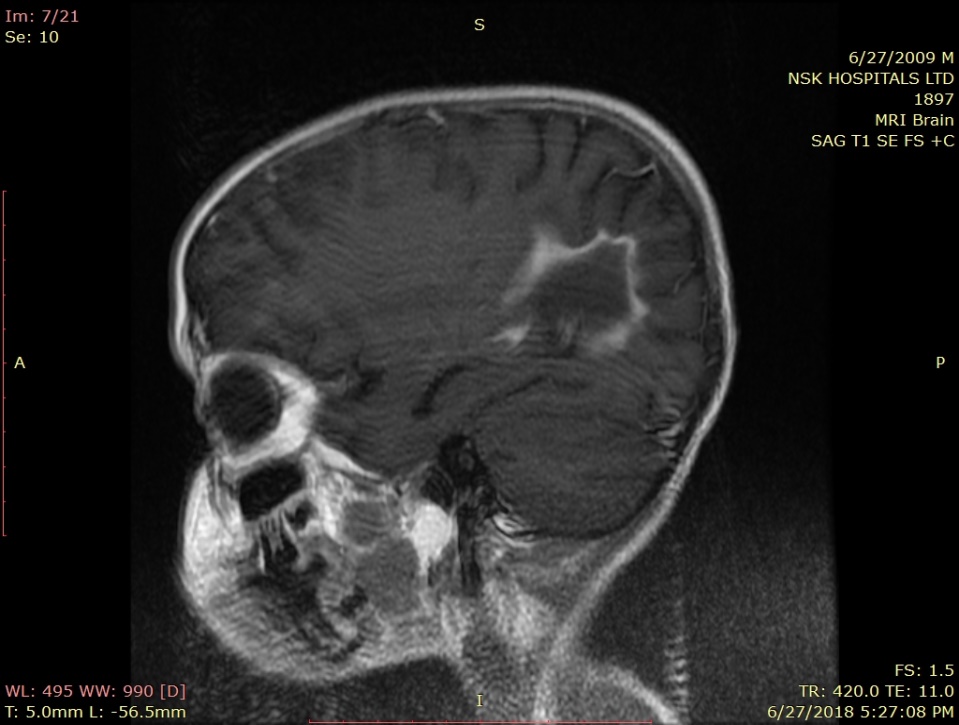


J K


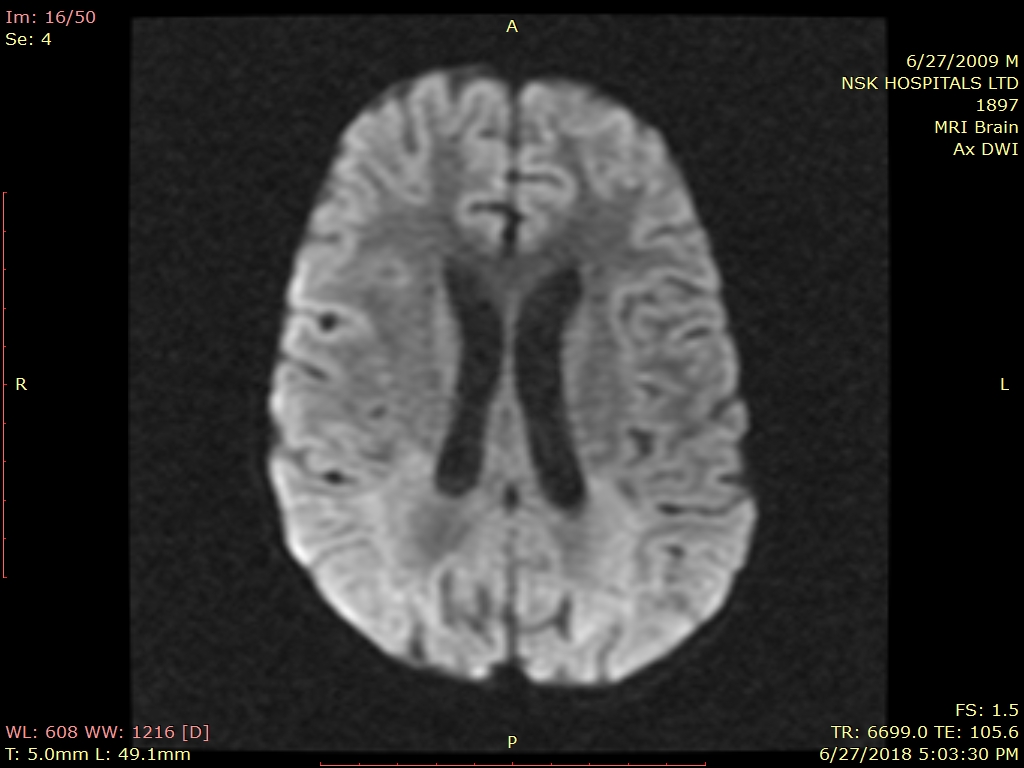

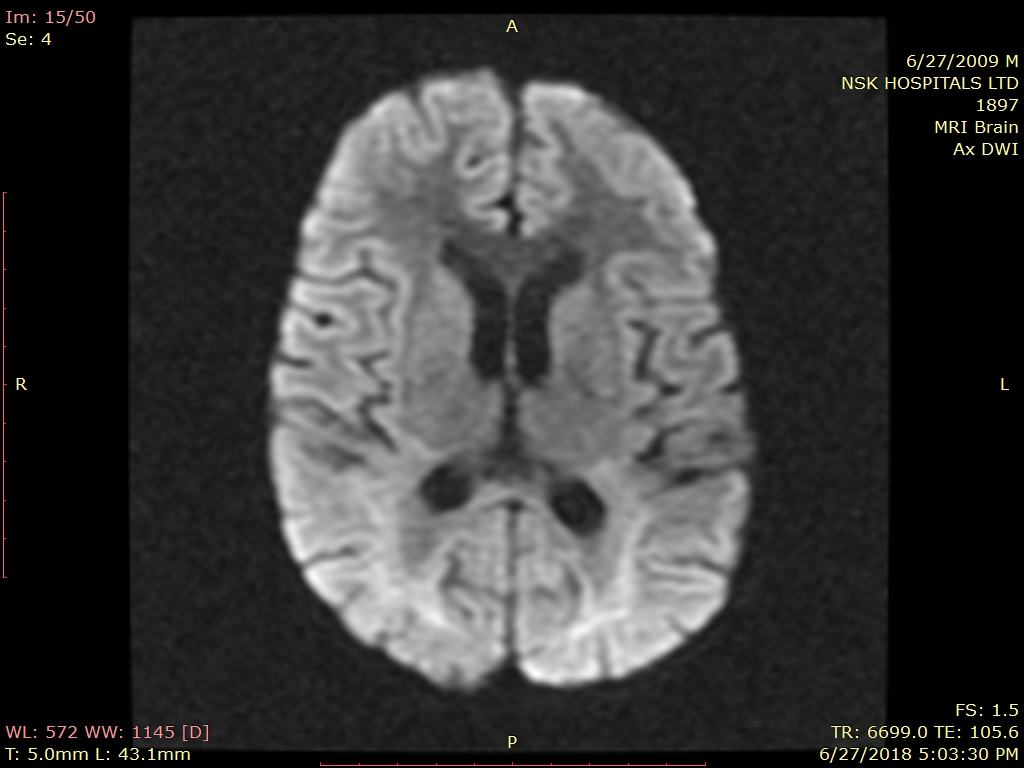


L M


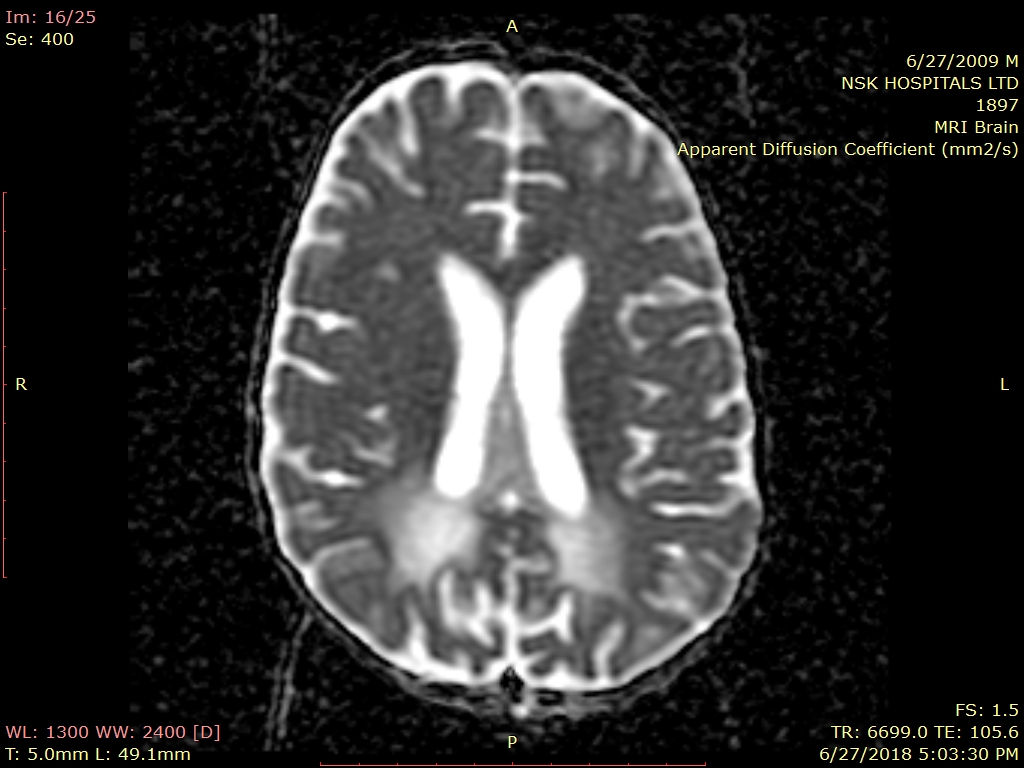

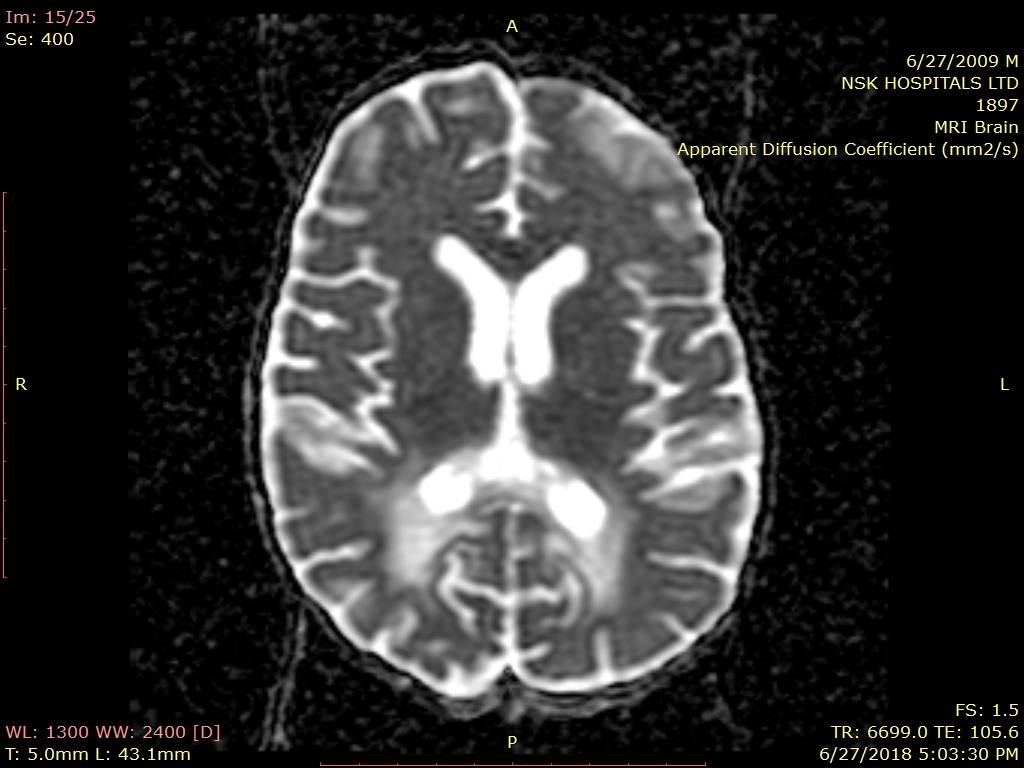

Supplement: Supplementary 2 — Figure S2: MRI of patient. S2A-S2B: Flair axial images. S2C-S2E: T2 weighted images. S2F-S2I: T1 contrast fat sat axial images. S2J-S2K: T1 contrast fat sat sagittal images. S2L-S2M: Diffusion Weighted Images (DWI). S2N-S2O: Apparent Diffusion Coefficient (ADC maps). MR sequences show bilateral symmetrical periventricular parietal white matter lesion which is hyperintense of Flair and T2WI and hypointense on T1 C+ fat sat images with peripheral intermediate zone enhancement. The lesion extends to the splenium of the corpus callosum, internal capsule, white matter tracks in the thalamus and inferiorly to the midbrain and pons. On DWI and ADC sequences, the intermediate zone of active demyelination shows restricted diffusion. Findings are consistent with X-linked adrenoleukodystrophy. [file 6148425.f2.docx]
